# Psychometric validation for a brand-new tool for the assessment of executive functions using 360° technology

**DOI:** 10.1038/s41598-023-35530-9

**Published:** 2023-05-27

**Authors:** Francesca Borgnis, Francesca Borghesi, Federica Rossetto, Elisa Pedroli, Mario Meloni, Giuseppe Riva, Francesca Baglio, Pietro Cipresso

**Affiliations:** 1 IRCCS Fondazione Don Carlo Gnocchi ONLUS, Via Capecelatro 66, Milan, Italy; 2 Department of Psychology, University of Turin, Turin, Italy; 3 Department of Geriatrics and Cardiovascular Medicine, IRCCS Istituto Auxologico Italiano, Milan, Italy; 4 Faculty of Psychology, eCampus University, Novedrate, Italy; 5 Humane Technology Lab, Università Cattolica del Sacro Cuore, Milan, Italy; 6 Applied Technology for Neuro-Psychology Lab, IRCCS Istituto Auxologico Italiano, Milan, Italy; 7 IRCCS Istituto Auxologico Italiano, Milan, Italy

**Keywords:** Neuroscience, Cognitive ageing, Cognitive neuroscience, Computational neuroscience, Diseases of the nervous system, Neurological disorders, Neurological disorders

## Abstract

EXecutive-functions Innovative Tool 360° (EXIT 360°) is an original 360° instrument for an ecologically valid and multicomponent evaluation of executive functioning. This work aimed to test the diagnostic efficacy of EXIT 360° in distinguishing executive functioning between healthy controls (HC) and patients with Parkinson’s Disease (PwPD), a neurodegenerative disease in which executive dysfunction is the best-defined cognitive impairment in the early stage. 36 PwPD and 44 HC underwent a one-session evaluation that involved (1) neuropsychological evaluation of executive functionality using traditional paper-and-pencil tests, (2) EXIT 360° session and (3) usability assessment. Our findings revealed that PwPD made significantly more errors in completing EXIT 360° and took longer to conclude the test. A significant correlation appeared between neuropsychological tests and EXIT 360° scores, supporting a good convergent validity. Classification analysis indicated the potential of the EXIT 360° for distinguishing between PwPD and HC in terms of executive functioning. Moreover, indices from EXIT 360° showed higher diagnostic accuracy in predicting PD group membership compared to traditional neuropsychological tests. Interestingly, EXIT 360° performance was not affected by technological usability issues. Overall, this study offers evidence that EXIT 360° can be considered an ecological tool highly sensitive to detect subtle executive deficits in PwPD since the initial phases of the disease.

## Introduction

Virtual reality-based (VR) evaluation can be considered a new promising paradigm for neuropsychological assessment, able to provide an ecological evaluation of everyday cognitive impairments predicting real-world performance^1^, compared to traditional paper-and-pencil or computerized neuropsychological batteries^2^. In the last few years, advances in 360° technology emerged as an interesting alternative approach to create VR content by recording familiar environments before, and then showing them to participants on a head-mounted display (HMD)^3,4^. Furthermore, 360° settings allow participants to be evaluated in virtual scenarios that they experience from a first-person perspective, overcoming some technical (e.g., high skills or cost) and clinical (e.g., vertigo, nausea) limitations of VR^1,5^. Borgnis and colleagues (2021) have developed EXecutive-functions Innovative Tool 360° (EXIT 360°), an original 360° instrument for an ecologically valid and multicomponent evaluation of executive functioning^6^. Participants are engaged in a “game for health” in which they are immersed in 360° household environments delivered via a conventional HDM. In these scenarios, subjects have to complete seven everyday subtasks designed to assess several aspects of executive functioning simultaneously and quickly. The need to develop EXIT 360° arose from the literature that showed the inability of traditional neuropsychological tests to detect executive impairments in everyday situations^7,8^. However, executive dysfunction constitutes a significant public health challenge in several psychiatric and neurological pathologies due to their high impact on personal independence (e.g., preparing meals, managing money, shopping, using a telephone), ability to work, educational success and social relationships^8–10^. Consequently, its ecologically valid assessment must be early and adequate to plan timely rehabilitation interventions^11,12^. Over the years, several studies have shown the feasibility and acceptability of VR-based tools in the early assessment of executive functioning in healthy controls and many psychiatric and neurologic pathologies (see review^13^). However, a recent systematic review (2022) has shown several psychometric issues in the available VR-based assessment tools for executive functioning due to limited studies on construct validity, discriminant validity, usability, and test re-test reliability^13^.

To date, EXIT 360° appears to be an innovative and interesting solution within the field of neuropsychological evaluation of executive functionality since preliminary studies involving healthy control subjects showed excellent results in construct validity^14^ and usability^15,16^. Firstly, EXIT 360° can be considered a usable, learn-to-use, clear, enjoyable, attractive, and friendly assessment tool, regardless of demographic characteristics (age and education) or technological expertise. Moreover, it appeared to be a fast, original, engaging, and challenging tool able to evaluate real-life impairments in several components of executive functioning (e.g., planning, decision-making, problem-solving, attention, visual searching, and working memory) with irrelevant adverse effects.

In light of these characteristics and psychometric proprieties, EXIT 360° could represent a promising tool for evaluating executive dysfunctions in several clinical populations, such as Parkinson’s Disease (PD). Indeed, in addition to the well-documented motor symptoms (e.g., bradykinesia, resting tremor, rigidity), people with PD frequently develop a wide range of non-motor symptoms (NMS) since the initial stages of the disease course, even before the onset of motor symptoms in the prodromal state^17–19^. Cognitive dysfunction represents one of the major clinical NMS of PD^17^, and executive dysfunction is the best-defined cognitive impairment in early-stage non-demented PD^20^, particularly affecting attention, planning, deduction, inhibition, capacity to elaborate a strategy, set-shifting (flexibility) and working memory^21^. As a result, patients have trouble in many goal-directed everyday activities, with negative implications for daily functioning (i.e., preparing meals, managing money, shopping, and work)^12,22,23^ and quality of life^23–25^. Interestingly, an increasing number of longitudinal studies suggested that early executive dysfunction is predictive of PD conversion in “PD with dementia”^26,27^. However, some studies showed that traditional standard assessment appeared not sensitive to the early detection of executive deficits^22^. Therefore, EXIT 360° could permit early detection of executive deficits and, consequently, identify patients at risk of developing dementia, providing early neurorehabilitative interventions^22,28^.

This study wants to deepen EXIT 360° diagnostic efficacy in distinguishing executive functionality between healthy subjects and patients with PD, also compared with traditional neuropsychological tests for executive functioning. It should be noted that the lack of discriminant validity constitutes a significant limitation in the use of a specific tool since the absence of information on diagnostic specificity and sensitivity in clinical populations makes it impossible to introduce them into clinical practice.

## Materials and methods

### Recruited sample

Thirty-six patients with Parkinson’s Disease (PwPD group) and 44 healthy controls (HC group) have been involved in this study. PwPD were consecutively recruited by an experienced neurologist at the Parkinson Center of IRCCS Fondazione Don Carlo Gnocchi ONLUS (FDG, Milan, Italy). HC were recruited among volunteers, family members and people participating in the public meeting. All participants have met the inclusion criteria: (a) Age between 18 and 90 years (b) education ≥ 5; (c) absence of overt dementia as determined by the Montreal Cognitive Assessment test^29^ (MoCA score ≥ 15.51, cut-off of normality), corrected for age and years of education according to Italian normative data^30^; (d) ability to provide written, signed informed consent. In addition, patients have met the following inclusion criteria: a) clinically established or probable Parkinson’s disease according to Movement Disorder Society (MDS)^31^; (b) mild to moderate disease staging, with scores < 3 on the Hoehn and Yahr scale; (c) suspected or confirmed deficits linked to executive functioning confirmed by documented neurological and/or neuropsychological evaluation. Exclusion criteria included (a) severe hearing or visual impairment that could compromise the assessment with EXIT 360°; (b) major systemic, psychiatric, or other neurological illnesses; (c) visual hallucinations or vertigo.

The study was approved by the “Fondazione Don Carlo Gnocchi-Milan” Ethics Committee. The neuropsychologist provided all participants with a complete explanation of the purpose and risk of the study before they signed the written informed consent based on the revised Declaration of Helsinki (2013).

All participants underwent a one-session evaluation at FDG that involved three main phases: (a) neuropsychological evaluation, (b) EXIT 360° session, and (c) usability assessment^32^.

### Neuropsychological evaluation

All subjects performed, in a clinical setting and before EXIT 360° completion, a neuropsychological evaluation of global and executive functioning, conducted by a trained neuropsychologist using conventional pencil–paper tests:

[a] Montreal cognitive assessment test (MoCA): a sensitive screening tool able to exclude the presence of global cognitive impairment.
[b] Integrated executive functions battery involving Trail Making Test^33^, phonemic verbal fluency task (F.A.S.)^34^, Stroop Test^35^, Digit Span Backward^36^, Frontal Assessment Battery (FAB)^37,38^, Attentive Matrices^39^ and Progressive Matrices of Raven (PMR)^40,41^ (for a detailed description of administered neuropsychological tests see^32^).

### EXIT 360° session

Each subject underwent an evaluation with EXIT 360°, preceded by a familiarization phase with the device and virtual environment to control any adverse effects (e.g., dizziness, nausea). Detailed characteristics and administration procedure of EXIT 360° have been recently described in a published study protocol^32^. Briefly, EXIT 360° is a 360°-based assessment tool for a complete assessment of executive functioning, engaging participants in a “game for health” delivered via smartphones, in which they have to perform seven subtasks (e.g., Unlock the Door; Choose the Person; Turn on the light) in 360° domestic environments (e.g., living room and bedroom). All participants sit on a swivel chair and wear the mobile-powered headset that allows for immersing in virtual environments explorable through the head’s movement as in real-life situations^42^. The test started when they heard, “*You are about to enter a house. Your goal is to get out of this house in the shortest time possible. To exit, you will have to complete a path and a series of tasks that you will encounter along your way. Are you ready to start?*”. To respond to each task, subjects had to choose between three or more alternatives by moving their head and positioning a small white dot that they saw in the headset on the answer for a few seconds. Participants had to perform all seven subtasks, obtaining one point for a wrong answer or two for a correct one. Overall, EXIT 360° allowed for the collection of Total Score (range 7–14) and Total Reaction Time (i.e., time spent to solve each of the 7 tasks, excluding instructions time. Then, the Total Reaction Time of EXIT 360° was calculated by summing the single reaction times).

### Usability assessment

All participants underwent a usability assessment of the technological tool using the System Usability Scale (SUS), a short questionnaire of 10 items on a 5-point scale from “completely disagree” to “strongly agree”^43,44^*.*

### Statistical analysis

Descriptive statistics included the frequencies, percentages, median and interquartile range (IQR) for categorical variables and the mean and standard deviation (SD) for continuous ones. The normality of data distribution was assessed using the Kolmogorov–Smirnov test. Demographics and clinical characteristics of the two groups (i.e., PD and HC) were compared using a t-test for independent samples (parametric or non-according to variables) or a chi-squared test for categorical variables. Moreover, ANOVA one-way between subjects was conducted to assess any differences between groups in traditional neuropsychological tests and EXIT 360° scores. Pearson’s correlation was applied to evaluate the possible relationship between the scores of the conventional neuropsychological tests and EXIT 360° scores (Total Score and Total Time). Finally, ROC curves evaluated all the tests’ specificity and sensitivity. Regarding system usability, Pearson’s correlation was conducted to compare EXIT 360° scores with the usability score. Moreover, ANOVA between subjects was performed to evaluate any differences in usability between the two groups. All statistical analyses were performed using Jamovi 1.6.7. Corrections for multiple comparisons were performed using the online calculator of False discovery rate correction for multiple comparisons. A *p*-value of < .05 was considered statistically significant.

Nonlinear stochastic approximation (i.e., machine learning) methods were used to compare the classification accuracy of traditional neuropsychological assessments versus the EXIT 360° indices for classifying participants into either the “Patients with PD” or “Healthy Controls” groups. Different machine learning algorithms were employed: Logistic Regression and Support Vector Machine algorithms. All these analyses were computed using Python 3.4.

Finally, we conducted an additional analysis dividing the PD sample into two groups according to performance on traditional neuropsychological (NPS) tests for executive functioning: Group PD_NPS + (i.e., patients with pathological/borderline performance in at least one NPS test) and Group PD_NPS- (i.e., patients that reported deficits in everyday activities linked to executive functioning—e.g., managing money or cooking—with a normal performance at NPS). We performed ANOVA between groups (PD_NPS + ; PD_NPI-; HC) to show any difference between the three groups in EXIT 360° and NPS scores. The results were reported in supplementary materials.

### Ethics approval

The study was conducted according to the guidelines of the Declaration of Helsinki and approved by “Fondazione Don Carlo Gnocchi—Milan” Ethics Committee.

### Informed consent statement

Informed consent was obtained from all subjects involved in the study.

## Results

### Participants

Table 1 reports the demographic and clinical characteristics of the whole sample (N = 80), divided into two groups (PwPD and HC). PwPD (*n* = 36) were predominantly female (M:F = 15:21) with a mean age of 68.7 (SD = 8.22, range = 53–84) and age of education = 13 (IQR = 6, range 5–18); HC were predominantly female (M:F = 18:26) with a mean age of 65.5 (SD = 13.8, range = 40–89) and age of education = 13 (IQR = 8.50, range 5–18). No significant differences between the two groups were detected in demographic characteristics and the global cognitive level assessed with the MoCA test. Moreover, all participants showed no cognitive impairment (MoCA score ≥ 15.51).

**Table 1:** Demographic and clinical characteristics of the whole sample.

	PwPD N = 36	HC N = 44	Group comparison (*p*-value)
Age (years, mean (SD))	68.7 (8.22)	65.5 (13.8)	.224
Sex (M: F)	15:21	18:26	.945
Age of education (years, median (IRQ))	13 (6)	13 (8.50)	.726
MoCA_adjusted score (mean (SD))	25.8 (2.41)	24.7 (2.72)	.082

*M* Male, *F* Female, *SD* Standard deviation, *IQR* Interquartile range, *n* Number, *MoCA* Montreal cognitive assessment, *PwPD* Patients with Parkinson’s Disease, *HC* Healthy controls.

### Traditional neuropsychological evaluation

Table 2 shows significant differences between the two groups in four neuropsychological tests of executive functioning. Specifically, HC achieved higher performance compared to PwPD in FAB score (F (1,78) = 27.81; *p* < .001) and PMR (F (1,78) = 7.82; *p* = .007). Moreover, HC group obtains better results compared to PwPD, in TMT-B (F (1,78) = 4.70; *p* = .033) and TMT-BA (F (1,78) = 5.32; *p* = .024). However, corrections for multiple comparisons showed the absence of differences between HC and PwPD in TMT-B (corrected *p*-value = .083) and TMT-BA (corrected *p*-value = .08).

**Table 2:** Comparison of scores at traditional neuropsychological tests.

	PwPD mean (SD)	HC mean (SD)	Group comparison (*p*-value)	Corrected *p*-value
Trail making test—A	32.68 (16.64)	30.59 (21.91)	.641	.801
Trail making test—B	117.28 (105.94)	78.52 (48.32)	**.033**	.083
Trail making test—B–A	85.5 (98.11)	49 (34.03)	**.024**	.08
Verbal fluency	37.81 (11.55)	38 (9.68)	.936	.936
Stroop test—errors	0.81 (3.1)	0.45 (0.76)	.463	.661
Stroop test—time	19.58 (13.15)	22.77 (13.41)	.289	.482
Digit span backward	4.47 (1.09)	4.52 (1.03)	.826	.918
Frontal assessment battery	15.71 (1.98)	17.52 (1.03)	** < .001**	**.001**
Attentive matrices	47.68 (7.44)	50.34 (6.57)	.094	.188
Progressive matrices of Raven	30.37 (4.04)	32.49 (2.73)	.**007**	**.035**

*SD* Standard deviation, *PwPD* Patients with Parkinson’s Disease, *HC* Healthy controls.

In bold, statistically significant value.

### EXIT 360°

Table 3 shows significant differences between the two groups in Total EXIT score (F (1,78) = 70.8; *p* < .001; η^2^*p* = .476) and Total Reaction time (F (1,78) = 52.8; *p* < .001; η^2^*p* = .404). Specifically, the HC group obtained a higher Total score compared to PwPD (mean = 12.5 ± 0.95) and completed the test in less time (mean = 484 ± 133.30).

**Table 3:** Comparison of scores at EXIT 360°.

	PwPD Mean (SD)	HC Mean (SD)	Group comparison (*p*-value)
Total EXIT score	10.2 ± 1.46	12.5 ± 0.95	** < .001**
Total reaction time	717.4 ± 153.98	484 ± 133.30	** < .001**

*SD* Standard deviation, *PwPD* Patients with Parkinson’s Disease, *HC* Healthy controls.

In bold, statistically significant value.

### Correlation between neuropsychological tests and EXIT 360°

Table 4 shows significant correlations (Pearson’s correlation) between traditional paper and pencil neuropsychological tests and the two scores of EXIT 360°.

**Table 4:** Correlation between EXIT 360° scores and neuropsychological assessment.

	PMR	AM	FAB	VF	DS	TMT-A	TMT-B	TMT-BA	ST-E	ST-T
EXIT-360° Total Score	.464**	.271**	.620**	.305*	.232*	− .309*	− .453**	− .424**	− .251*	**− **.218
EXIT-360° Total Time	− .333*	**− **.209	− .433**	**− **.084	**− **.009	.170	.477**	.489**	.199	.139

*PMR* Progressive matrices of raven, *AM* Attentive matrices, *FAB* Frontal assessment battery, *VF* Verbal fluency, *DS* Digit span, *TMT-A* Trail making test-part A, *TMT-B* Trail making test-part B, *TMT-BA* Trail making test-part B–A, *ST-E* Stroop test-errors, *ST-T* Stroop test-time.

In bold, statistically significant scores.

*p < .05; **p < .001.

### Classification of healthy controls or clinical group

**Figure 1:**
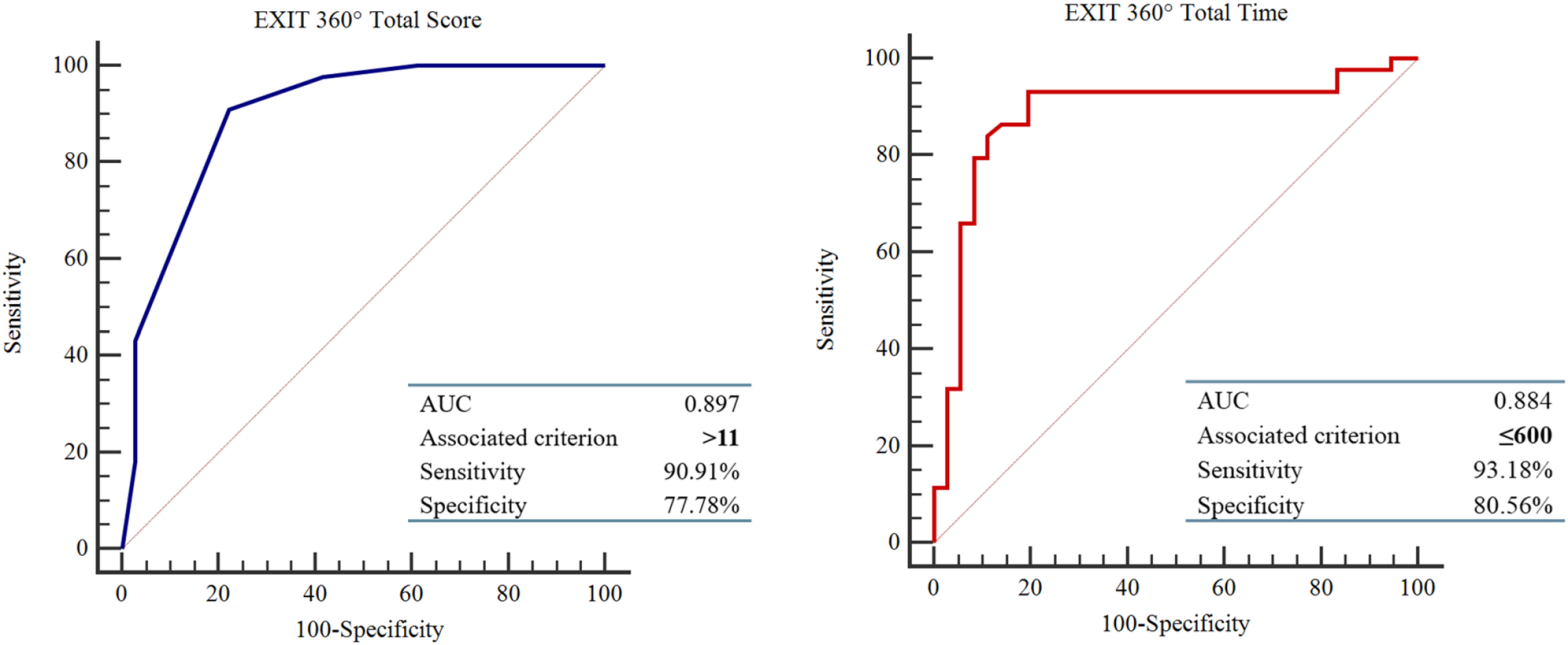
ROC Curve—EXIT 360° Total Time and Total Score.

The performance of the classifiers was evaluated by carrying out a relative operating characteristic (ROC) analysis. The area under the ROC curve (AUC) provides a single measure of overall prediction accuracy. Specifically, ROC curves investigated the diagnostic accuracy of EXIT 360° showing that (Fig. 1):

- EXIT Total Score ≤ 11 could accurately discriminate HC and PwPD groups, with high sensitivity (90.91%) and specificity (77.78%) (AUC = 0.897—excellent accuracy value).
- EXIT Total Time > 600 could accurately discriminate HC and PwPD groups, with high sensitivity (93.18%) and specificity (80.56%) (AUC = 0.884—excellent accuracy value).

**Figure 2:**
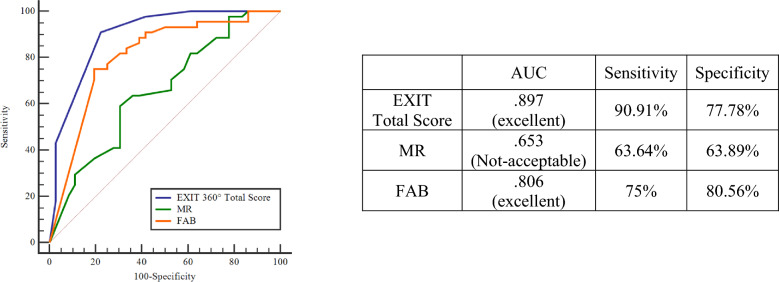
ROC Curve—Comparison between EXIT 360° Total Score and neuropsychological tests. *MR* Raven’s progressive matrices, *FAB* Frontal assessment battery.

Further analyses showed EXIT Total Score ≤ 11 can discriminate between HC and PwPD with better overall prediction accuracy, sensitivity, and specificity than MR (DeLong test—*p* < .001) and FAB (DeLong test—*p* = .04) scores (Fig. 2).

**Figure 3:**
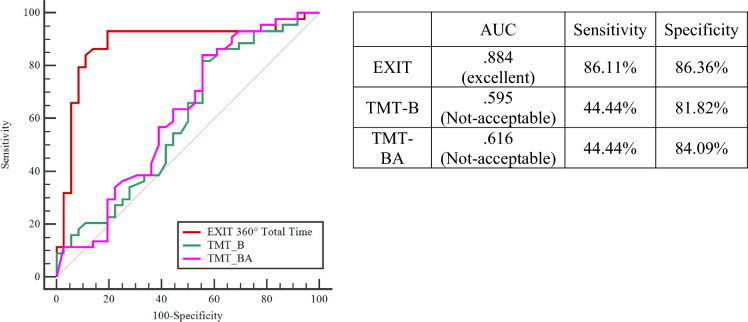
ROC Curve—Comparison between EXIT 360° Total Time Score and neuropsychological tests. *TMT* Trail making test.

Moreover, EXIT Total Time > 600 allows for discriminating between HC and PwPD with better overall prediction accuracy, sensitivity, and specificity than TMT-B and TMT-BA (DeLong test—*p* < .001) scores (Fig. 3).

In addition, nonlinear stochastic approximation methods confirmed the ROC analyses, showing an excellent accuracy of EXIT 360° scores in discriminating HC and PwPD. Results showed a precision (i.e., the proportion of true positives among all the instances classified as positive) between 61 and 65% for the conventional neuropsychological assessment of executive functions (Table 5 panel A), while it ranged from 79 to 86% for EXIT 360° (Table 5 panel C) and from 80 to 90% for traditional battery and EXIT 360° together (Table 5 panel B).

**Table 5:** Leave one out cross-validation (LOOCV) for the traditional neuropsychological tests [A], the indices of EXIT-360° and the traditional neuropsychological tests [B], and the indices of EXIT-360° [C].

Methods	AUC	CA	F1	Precision	Recall
[A] Traditional neuropsychological tests					
k-nearest neighbors (kNN)	0.67	0.61	0.61	0.61	0.61
Logistic regression	0.67	0.61	0.61	0.61	0.61
Naive bayes	0.68	0.65	0.65	0.65	0.65
Support vector machine (SVM)	0.68	0.64	0.63	0.63	0.64
[B] EXIT-360° and Traditional Neuropsychological tests					
k-nearest neighbors (kNN)	0.86	0.80	0.80	0.80	0.80
Logistic regression	0.93	0.90	0.90	0.90	0.90
Naive bayes	0.85	0.80	0.80	0.80	0.80
Support vector machine (SVM)	0.90	0.81	0.81	0.81	0.81
[C] EXIT-360°					
k-nearest neighbors (kNN)	0.86	0.79	0.79	0.79	0.79
Logistic regression	0.91	0.85	0.85	0.85	0.85
Naive bayes	0.91	0.83	0.83	0.83	0.83
Support vector machine (SVM)	0.91	0.85	0.85	0.86	0.85

AUC (Area under the ROC curve) is the area under the classic receiver-operating curve; CA (Classification accuracy) represents the proportion of the examples that were classified correctly; F1 represents the weighted harmonic average of the precision and recall (defined below); Precision represents a proportion of true positives among all the instances classified as positive. In our case, the proportion of conditions correctly identified; Recall represents the proportion of true positives among the positive instances in our data.

**Figure 4:**
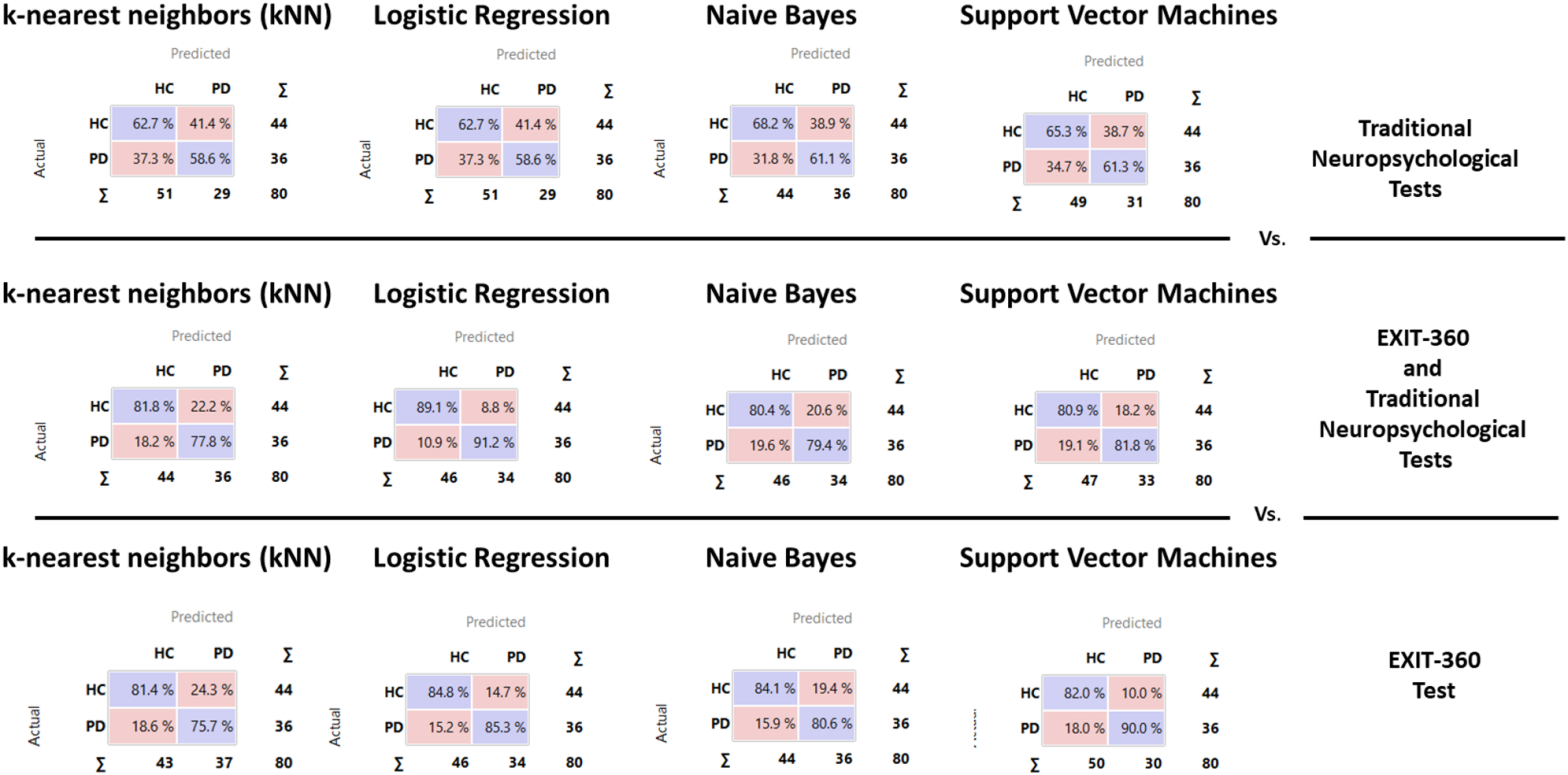
Classification of HC or PwPD. The diagonal values (i.e., purple boxes) represent the elements for which the predicted group is equal to the true group, while of-diagonal elements are those that are mislabeled by the classifier. Logistic Regression and Support Vector Machine algorithms demonstrated that EXIT-360° has a higher capability in predicting PD Group membership with respect to traditional neuropsychological tests of executive functioning.

Interestingly, all machine learning algorithms showed that the indices from EXIT 360° had a higher capability in predicting PD Group membership compared to traditional neuropsychological tests of executive functioning (Fig. 4).

### Usability

The comparison between the PwPD and HC showed the absence of a significant difference in usability score (F (1,78) = .415; *p* = .521). Indeed, PwPD provided a mean score of 77.3 ± 9.30, while HC showed a mean score of 75.7 ± 12.41. Both scores indicate a satisfactory level of usability, according to the scale’s score acceptability ranges (cut off = 68) and adjective ratings (included between “good” and “excellent”). Moreover, Pearson’s correlation showed no significant relationship between the total usability score and, respectively, EXIT 360° Total score (*p* = .711) and EXIT 360° Time score (*p* = .560).

## Discussion

This work aimed to determine whether EXIT 360° could integrate the conventional paper-and-pencil neuropsychological assessment in PD, providing a more ecologically valid evaluation of executive functions. Firstly, we examined the performance of PwPD and HC by comparing the traditional neuropsychological battery with the EXIT 360° to evaluate its efficacy in detecting executive deficits. Correlations between performances on EXIT 360° and paper-and-pencil tests were also investigated. Finally, we looked into the predictive validity of EXIT 360° scores in discriminating PwPD from HC in executive functioning. All subjects were in a relatively well-preserved clinical state. However, the neuropsychological assessment of executive functions showed differences between patients and HC in two tests (FAB and PMR). Correlation analyses indicated that neuropsychological tests correlate significantly with EXIT 360° scores, supporting a good convergent validity^14^. Therefore, EXIT 360° can be considered a tool able to detect several components of executive functioning, including cognitive flexibility, inhibition control, sustained and selective attention and processing speed.

As regards analyses on EXIT 360°, our main findings revealed significantly different performances between patients and cognitively healthy participants in both EXIT 360° scores. Specifically, PD patients, compared with HC, made more errors in completing the subtasks of EXIT 360° and took longer to conclude the test (i.e., a slower processing speed). These results showed that EXIT 360° is an ecological tool highly sensitive to executive impairment in PD since the mild-to-moderate stage of PD (Hoehn e Yahr scores < 3) when motor symptoms outweigh the cognitive ones. This assumes considerable importance in clinical practice since executive deficits in the early stage of PD are predictive of the conversion to dementia^20,45–47^, with a negative impact on everyday functioning^8,24,25^. Therefore, identifying patients with a potentially higher risk of dementia appears to be a priority to plan an early and individualized cognitive rehabilitation treatment^22,28^.

Interestingly, our results align with a previous study on PwPD that showed the efficacy of a VR-based instrument, VMET, in evaluating executive impairments, which had not been fully acknowledged by traditional neuropsychological evaluations^22^. It is well known that the most crucial issues of the traditional neuropsychological tests are the lack of ecological validity and the ability to measure only one specific component of executive functions without reflecting an accurate and complex picture of a patient’s executive status^7,8,48^. For this reason, patients with presumed executive deficits can perform similarly to HC on traditional neuropsychological tests yet encountering difficulties in real-world situations^22^. In this context, the technology 360° may be used to offer a new paradigm in which patients are active participants within an ecological virtual world^1,49^ in which it is possible to simulate life-like challenges that reproduce everyday situations. In this framework, EXIT 360° has proved to be an innovative tool for detecting executive dysfunction through a function-led approach that combined experimental control with an engaging real-world background.

In addition, results obtained from ROC curve analyses clearly indicated the potential of EXIT 360° scores (accuracy and completion time) in distinguishing between PwPD and HC in terms of executive functioning. Specifically, a total score of ≤ 11 allows for accurate (AUC = .897) discrimination of PwPD compared to HC with high sensitivity and specificity. The same results appear considering the total time score, where a value > 600 allows for accurately (AUC = .884) discriminating between patients and controls in terms of processing speed. Moreover, ROC curve analyses allowed us to demonstrate the ability of EXIT 360° Total Score and EXIT 360° Time Score to discriminate between HC and PwPD with better overall prediction accuracy, sensitivity, and specificity than traditional paper-and-pencil neuropsychological assessment for executive functioning. Conventional neuropsychological tests, such as FAB, are stronger tools when the cognitive deficit is overt. In line with the previous evidence, EXIT 360° would be a tool more sensible in the early stage of executive impairments since it collected some ecological aspects that impact everyday functioning^22,50,51^.

These findings were also confirmed by the higher diagnostic accuracy in machine learning classification of participants to the clinical or non-clinical conditions (when using indices from EXIT 360°) with respect to those from neuropsychological assessments. These robust findings demonstrated the efficacy of EXIT 360° for detecting impairment of several components of executive functioning at an early clinical stage of PD. Therefore, EXIT 360° can be considered an ecological tool highly usable for prompt diagnosis of executive dysfunction and early enrolment of patients in targeted rehabilitation.

Interestingly, machine learning analyses have also suggested that integration between neuropsychological tests and EXIT 360° could allow better classification accuracy, with precision ranging between 80 and 90%. This result supported the potentiality of EXIT 360° to integrate the traditional neuropsychological assessment of EFs in PD with a more ecologically valid assessment.

Overall, our results confirm that the 360° technology may play a key role in neuropsychological assessment, in accordance with previous studies^5^. In particular, our findings follow previous evidence on the 360° version of the Picture Interpretation Test (PIT) ability to detect executive dysfunction in active visual perception, typical of PwPD compared to HC. EXIT 360° can be considered an evolution of the PIT 360°, allowing to evaluate several components of executive functioning in an ecological context. The multicomponent aspect appears critical in the clinical evaluation of PwPD since several studies have shown the presence of several executive impairments in PD, such as planning, attention, working memory, set-shifting, dual-task performance, inhibitory control, and decision making, including social–cognition abilities^9,13,52,53^. In addition, EXIT 360° reproduces everyday domestic settings, such as the kitchen, bedrooms, living room, and landing, allowing an evaluation of the executive impairments in the environment most experienced by the subject, with wide implications also in terms of rehabilitation. This feature is peculiar to EXIT 360° since all technological-based tools for evaluating executive functions in PD have involved only a few everyday life scenarios, especially supermarkets but never domestic environments^13^. Finally, in line with a previous study^16^, EXIT 360° obtained good to excellent usability score and the absence of correlation between total usability score and EXIT 360° indexes. Therefore, low performances of participants did not depend on technological usability.

While the current study’s findings are promising, some limitations and future perspectives should be considered. Firstly, to fully evaluate the potentiality of EXIT 360° as a new screening tool for executive functions, future studies are needed to assess its test–retest and inter-rater reliability. Additionally, although participants do not need to move in the environment, they must explore the environment by moving their heads; therefore, it cannot be excluded that EXIT 360° could involve other cognitive domains, such as motor representation and programming. Future studies should investigate this aspect by including measures of motor functioning in a regression analysis along with executive functions. Moreover, it will be important to investigate the value of EXIT 360° in detecting executive impairments in other neurological populations known to have executive dysfunctioning, such as Multiple Sclerosis, Mild Cognitive Impairments and Alzheimer’s Disease. Finally, it will be of fundamental importance to develop and validate a parallel form of EXIT 360° to make possible a short-term revaluation in a rehabilitation process.

In conclusion, this study offers evidence that a more ecologically valid evaluation of executive functions is more likely to detect subtle executive deficits in PD patients. EXIT 360° captures early executive dysfunctions of PwPD with better accuracy than the traditional neuropsychological assessment. In this context, we think EXIT 360° has a great potentiality in integrating the traditional paper-and-pencil neuropsychological assessment in PD, with a more ecologically valid assessment of executive functions. This innovative 360°-based instrument, easily accessible and clinically usable, can radically transform patients' and clinicians' assessment experience. Firstly, the times for evaluating executive functionality will be drastically reduced since EXIT 360° lasts at most 15 min. In addition, neurologists and neuropsychologists can get ecologically-valid multicomponent evaluations of executive functioning in PD, collecting the real executive status of patients. The ecological assessment will allow tailoring rehabilitation to the everyday subject’s needs. As previously said, a timely intervention on executive dysfunction in early-stage non-demented PD could minimize the impact of this significant clinical non-motor symptom, increasing the patient's daily functioning and quality of life^20,21^. Interestingly, as it was designed, EXIT 360° could also be used by a streaming platform that would allow to carry out the remote assessment, overcoming the social distancing limits.

## Supplementary Information

Supplementary Information.

## Data Availability

The tool and datasets generated and/or analysed during the current study are available in the Zenodo repository, https://doi.org/10.5281/zenodo.7006781.
