# Supplementary material for: Psychometric validation for a brand-new tool for the assessment of executive functions using 360° technology
Source: Sci Rep. 2023 May 27;13:8613. doi: 10.1038/s41598-023-35530-9 (PMC10224951; doi:10.1038/s41598-023-35530-9)
Supplement: Supplementary file 1 — Supplementary Information. [file 41598_2023_35530_MOESM1_ESM.docx]

**Supplementary Material**

PD sample was divided into two groups according to performance on neuropsychological (NPS) tests for executive functioning: Group PD_NPS+: patients with pathological/borderline performance in at least one NPS test; Group PD_NPS-: patients that reported deficits in everyday activities linked to executive functioning (e.g., managing money or cooking) with a normal performance at NPS.

Supplementary Table 1 reports the demographic and clinical characteristics of the whole sample, divided into three groups (PD_NPS+, PD_NPS- and HC). No significant differences between groups were detected in demographic characteristics (age, sex and education).

|  | **PD_NPS+**  **N=18** | **PD_NPS-**  **N=18** | **HC**  **N=44** | **Group Comparison (p-value)** |
| --- | --- | --- | --- | --- |
| **Age** (years, mean (SD)) | 70.44 (6.61) | 67.00 (9.44) | 65.52 (13.77) | 0.324 |
| **Sex** (M: F) | 8:10 | 7:11 | 18:26 | 0.942 |
| **Age of education** (years, median (IRQ)) | 13 (5) | 13 (4.75) | 13(8.50) | 0.178 |

**Supplementary Table 1.** Demographic and clinical characteristics of the whole sample. M = male; F = female; SD = standard deviation; IQR = interquartile range; *n* = number; PD=patients with Parkinson’s Disease; NPS=neuropsychological tests HC= Healthy controls

Then, we conducted ANOVA between groups (PD_NPS+; PD_NPI-; HC) to show any difference between the three groups in EXIT 360° and NPS scores. The results are reported in supplementary Table 2 and in Supplementary Figure 1. The ANOVA shows statistically significant differences in EXIT 360° scores and in NPS tests (i.e., FAB, TMT, PMR, AM, VF and ST-Time), except for Digit Span Backward (p=.149), Stroop Test – Errors (p=.98) and Trail Making Test part-A (p=.137). Post-hoc ANOVA comparisons after multiple corrections (Tukey) showed that only EXIT 360° Total score was statistically significantly different between Group PD_NPS- and HC (mean difference=-1.50, p<.001).

Figure 1 shows the distribution of the EXIT 360° scores and two NPS tests (FAB for accuracy score and TMT-B for processing speed score) obtained by three groups. On each plot, the normality cut-offs, represented by dashed red lines, were indicated according to Italian normative papers for NPS or ROC and nonlinear analysis described in the manuscript for EXIT 360° scores. The results show as EXIT 360° scores allowed to discriminate between PD and HC in terms of executive functioning, even considering separately patients with pathological/borderline performance at NPS tests and patients who complain about difficulties in everyday activities not yet collected by the NPS tests.

|  | **PD_NPS+**  Mean (SD) | **PD_NPS-**  Mean (SD) | **HC**  Mean (SD) | **Group Comparison**  **(p-value)** | **Post-Hoc Comparison** |
| --- | --- | --- | --- | --- | --- |
| EXIT 360°  Total Score | 9.44 (1.04) | 11 (1.41) | 12.5 (.95) | <.001 | PD_NPS+< PD_NPS-<HC |
| EXIT 360°  Total Time | 767 (140.12) | 667.78 (154.82) | 484 (133.30) | <.001 | PD_NPS+>HC  PD_NPS->HC |
| TMT - A | 39 (19.32) | 26.33 (10.58) | 30.59 (21.91) | .137 | - |
| TMT – B | 167.39 (128.94) | 67.17 (34.13) | 78.52 (48.32) | <.001 | PD_NPS+> PD_NPS-  PD_NPS+>HC |
| TMT - BA | 128 (123.09) | 43 (29.01) | 49 (34.03) | <.001 | PD_NPS+> PD_NPS-  PD_NPS+>HC |
| VF | 33.12 (12.63) | 42.5 (8.27) | 38 (9.68) | .025 | PD_NPS+< PD_NPS- |
| ST – E | 1.53 (4.32) | .08 (0.30) | .45 (.76) | .098 | - |
| ST - T | 25.28 (15.69) | 13.88 (6.44) | 22.77 (13.41) | .019 | PD_NPS+> PD_NPS-  PD_NPS->HC |
| DS | 4.13 (1.07) | 4.81 (1.03) | 4.52 (1.03) | .149 | - |
| FAB | 14.78 (2.22) | 16.64 (1.13) | 17.52 (1.03) | <.001 | PD_NPS+< PD_NPS-  PD_NPS+<HC |
| AM | 44.47 (8.18) | 50.89 (5.03) | 50.34 (6.57) | .005 | PD_NPS+< PD_NPS-  PD_NPS+<HC |
| PMR | 29.16 (4.55) | 31.57 (3.12) | 32.49 (2.73) | .002 | PD_NPS+<HC |

**Supplementary Table 2.** Comparison of scores at EXIT 360° scores and traditional neuropsychological tests. SD = standard deviation; PD=patients with Parkinson’s Disease; HC= Healthy Controls. PMR= Progressive Matrices of Raven; AM= Attentive Matrices; FAB= Frontal Assessment Battery; VF= Verbal Fluency; DS= Digit Span; TMT-A= Trail Making Test – part A; TMT-B= Trail Making Test – part B; TMT-BA= Trail Making Test – part B-A; ST-E= Stroop Test – Errors; ST-T= Stroop Test – Time.


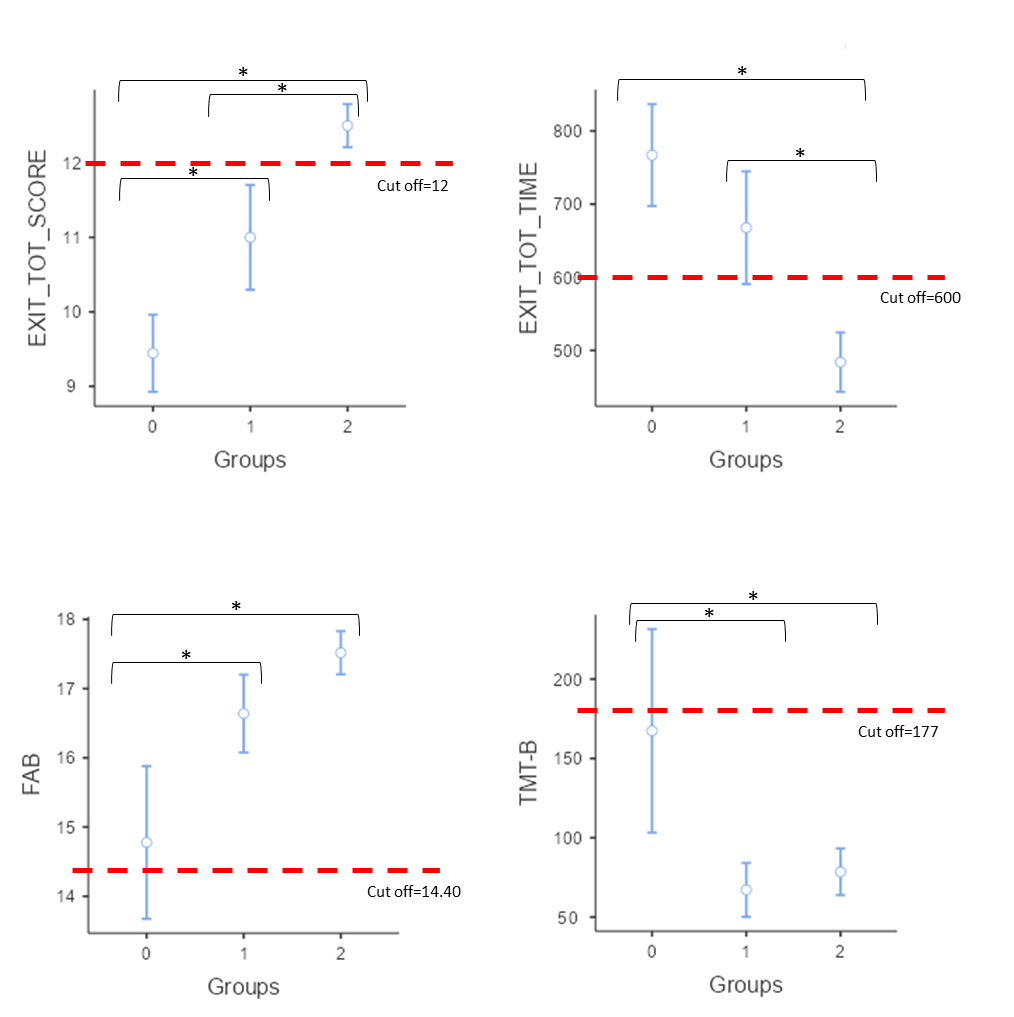


Supplementary Figure 1. Distribution of EXIT 360° scores and two NPS tests divided into the three groups. *=p<.05; dashed red line indicates normal cut-off of tests. Cut-off for NPS tests derives from Italian normative data (Appollonio et al. 2005; Giovagnoli et al., 1996). The cut-off for EXIT 360° scores derives from ROC curve analysis and nonlinear stochastic approximation described in the manuscript.
